# Activation of JNK and p38 in MCF-7 Cells and the In Vitro Anticancer Activity of *Alnus hirsuta* Extract

**DOI:** 10.3390/molecules25051073

**Published:** 2020-02-27

**Authors:** Mina Ryu, Chung Ki Sung, Young Jun Im, ChangJu Chun

**Affiliations:** College of Pharmacy, Chonnam National University, Gwangju 61186, Korea; pegasus721@naver.com (M.R.); chksung@jnu.ac.kr (C.K.S.)

**Keywords:** *Alnus hirsuta*, diarylheptanoid, MCF-7 cells, MAPK, JNK, p38, ROS, apoptosis, cell cycle

## Abstract

JNK and p38 are important mitogen-activated protein kinases (MAPKs) that respond to stress stimuli. The stress-activated MAPKs associated with apoptotic cell death play vital roles in mammalian cells. *Alnus hirsuta*, which contains abundant diarylheptanoids derivatives, is a valuable medicinal plant. The CHCl_3_ extract (AHC) containing platyphyllenone (**1**) and platyphyllone (**3**) as main compounds showed in vitro anticancer effects. We report the biological activities of *A. hirsuta* extract associated with the regulation of apoptosis and JNK and p38 in MCF-7 breast cancer cells. Levels of phospho-JNK and phospho-p38 by AHC treatment were evaluated by enzyme-linked immunosorbent assay (ELISA). ROS production, apoptotic effect, and DNA contents of the cells were measured by flow cytometry. The two diarylheptanoids **1** and **3** and the AHC extract exhibited cytotoxic effects on MCF-7 cells in MTT assay, with IC_50_ values of 18.1, 46.9, 260.0 μg/mL, respectively. AHC induced ROS generation and elevated the endogenous levels of phospho-JNK and phospho-p38. AHC resulted in apoptosis and cell cycle arrest. We suggest that the antitumor effect of *A. hirsuta* extract is achieved by apoptosis promotion and cell cycle arrest mediated by the activation of JNK and p38 signaling pathway via ROS generation.

## 1. Introduction

Breast cancer is a disease caused by unregulated proliferation of malignant cells in the mammary epithelial tissue [1]. The etiology of breast cancer is still not fully understood, however, it is believed that genetic and hormonal factors play an important role in the development of the disease [2,3]. Breast cancer is highly heterogeneous, encompassing a group of genetically and epigenetically distinct diseases exhibiting diverse clinical features. 84 subtypes of breast cancer including variants of MCF-7 cells are categorized by phenotypes of three receptors: estrogen receptor (ER), progesterone receptor (PR), and human epidermal growth factor receptor-2 (HER2; also known as ErbB2) [4]. ER, PR, and HER2 are the hormone receptors that control the growth of breast cancer cells, accompanied by the epidermal growth factor receptor (EGFR) [5]. Variety of tailored approaches for the clinical goals has been developed.

Mitogen-activated protein kinases (MAPKs) are Ser/Thr kinases that convert extracellular stimuli into a wide range of cellular responses. The MAPK families play a pivotal role in complicated cellular programs such as proliferation, differentiation, development, transformation, and apoptosis [6]. In mammalian cells, three MAPK families have been distinctly characterized: extracellular signal-regulated kinase (ERK), C-Jun N-terminal kinase/also termed stress-activated protein kinase (JNK/SAPK) and p38 MAPK. ERK pathway is mainly involved in growth, differentiation, and development, while JNK and p38 MAPK pathways are principally involved in inflammation, apoptosis, growth, and differentiation [7,8].

The stress-activated MAPKs, p38 and JNK, play vital roles, particularly in breast cancer and lung cancer [9,10]. Although, the role of JNK and p38 in cancer is complicated and controversial [7], the expression levels of the proteins tend to increase in the breast cancer tissue compared to normal tissues. JNK and p38 are regarded as important prognostic factors in breast cancer [11,12,13]. JNK signaling is required for normal mammary gland development and it has a suppressive role in tumorigenesis [11]. The possibility has been suggested that upregulation of p38 activity can inhibit the onset of breast tumors, inhibit tumor metastasis, and be an implicit parameters for sensitization to tumor suppression and apoptosis [14,15,16]. The understanding of the important functions of p38 in the process of differentiation and cell death has recently grown and is now relatively established [17]. ERK plays a crucial role in most cellular functions, and also can mediate different antiproliferative events, such as apoptosis, autophagy and senescence depending on the cell type and stimulus [18,19,20]. There is growing evidence indicating that ROS can stimulate the activation of ERK, JNK, and p38MAPK [21].

Apoptosis, a protective mechanism to eliminate aberrant cells, is a vital programmed cell-death mechanism to preserve tissue homeostasis. Apoptosis has been recognized as a key form of cell death in biological processes and various pathologies. ROS play a key role in cellular signaling control of redox process during apoptotic mechanism [22]. The perception that apoptosis and its related genes have a decisive influence on malignant phenotype is generally acknowledged. Apoptosis contributed to the high rate of cell loss in malignant tumors [23]. Targeting apoptosis is one of the effective strategies in cancer therapy, and the elucidation of mechanisms related to the signal transduction pathways is of medical significance. 

*Alnus*, an important genus of the Betulaceae, contains abundant diarylheptanoids as its characteristic constituents. Barks of *Alnus hirsuta* have been used as a folklore medicine for various diseases such as liver diseases, diabetes and bronchial disorders in Korea. Diarylheptanoids contain of aromatic and aliphatic skeletons in their skeleton, that is 1,7-diphenylheptane frameworks. Hirsutanone, hirsutanolol, oregonin, and alnusonol are the representative diarylheptanoids from the genus *Alnus* [24,25]. Previous studies have reported that *Alnus* plants possess a variety of terpenoids, flavonoids, polyphenols, steroids, and tannins, besides. Numerous bioactivities of genus *Alnus* were reported, including hepato-protective, antioxidant, and antitumor activities [26,27,28,29,30,31].

Enzyme-Linked Immunosorbent Assay (ELISA) is one of the high precision quantitative solid-phase immunoassays most commonly used for the assessment of antigen composition and cellular functions in analytical biochemistry. The solid-phase immunoassays such as ELISA provide a highly sensitive means to measure the presence of antigen in defined and homogeneous samples, for instance purified proteins in buffer. The approaches allow the verification of antigen presence in undefined heterologous biological samples such as cell lysates, tissue culture supernatants, blood, and other clinical samples as well as [32].

ELISA was conceptualized in the 1960s and developed and early used during the 1970s and 1980s. The invention of ELISA led to the development of enzyme labels in immunoassays. Today, the immunoassay principle with an enzyme has been used as the reporter label for routine measurements of numerous analytes in patient samples in automated instruments in medical laboratories around the world. The method was considered greatly significant as it was based on the principle of immunoassay using enzymes rather than radiation as a reporter label in the early days of development [33].

Natural product research for the discovery of new compounds possesses the potential for the development of therapeutics that can treat a variety of diseases, including cancer. *Alnus hirsuta* was known to contain abundant diarylheptanoids [24,30]. Current study was aimed at evaluating the effects of *A. hirsuta* on human cancer cells and elucidating the underlying mechanism. We applied sandwich type ELISA to illuminate the apoptotic pathway.

We implemented preferentially cytotoxicity assay to estimate the antiproliferative effect of *A*. *hirsuta* extract against three different types of cancer cell lines. We performed additionally the in vitro biological assays for the evaluation of anticancer effect of *A. hirsuta* extract on MCF-7 cells, which shown the highest antiproliferative activity. The efficacy of *A. hirsuta* extract on cell viability assessed by MTT assay was established by apoptotic and cell cycle arrest effect. Activated apoptotic pathway in MCF-7 cells treated with the extract was presented by examining the levels of JNK, p38, and inflammation-related factors along with the level of ROS generated in the cells. Treatment of the *A. hirsuta* extract reduced the cell viability of MCF-7 cells. We observed that ROS production level increased in the cells by treatment of the extract. The extract activated the pathway of p38 the most and induced cell cycle arrest and apoptosis of MCF-7 cells.

## 2. Results

### 2.1. Solvent Extracts of Alnus hirsuta and the Constituents of the AHC Extract

**Figure 1:**
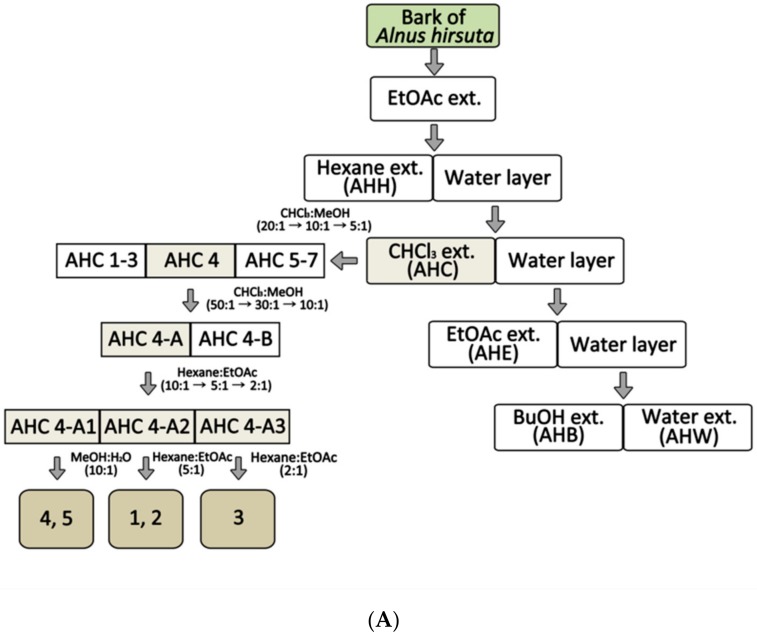

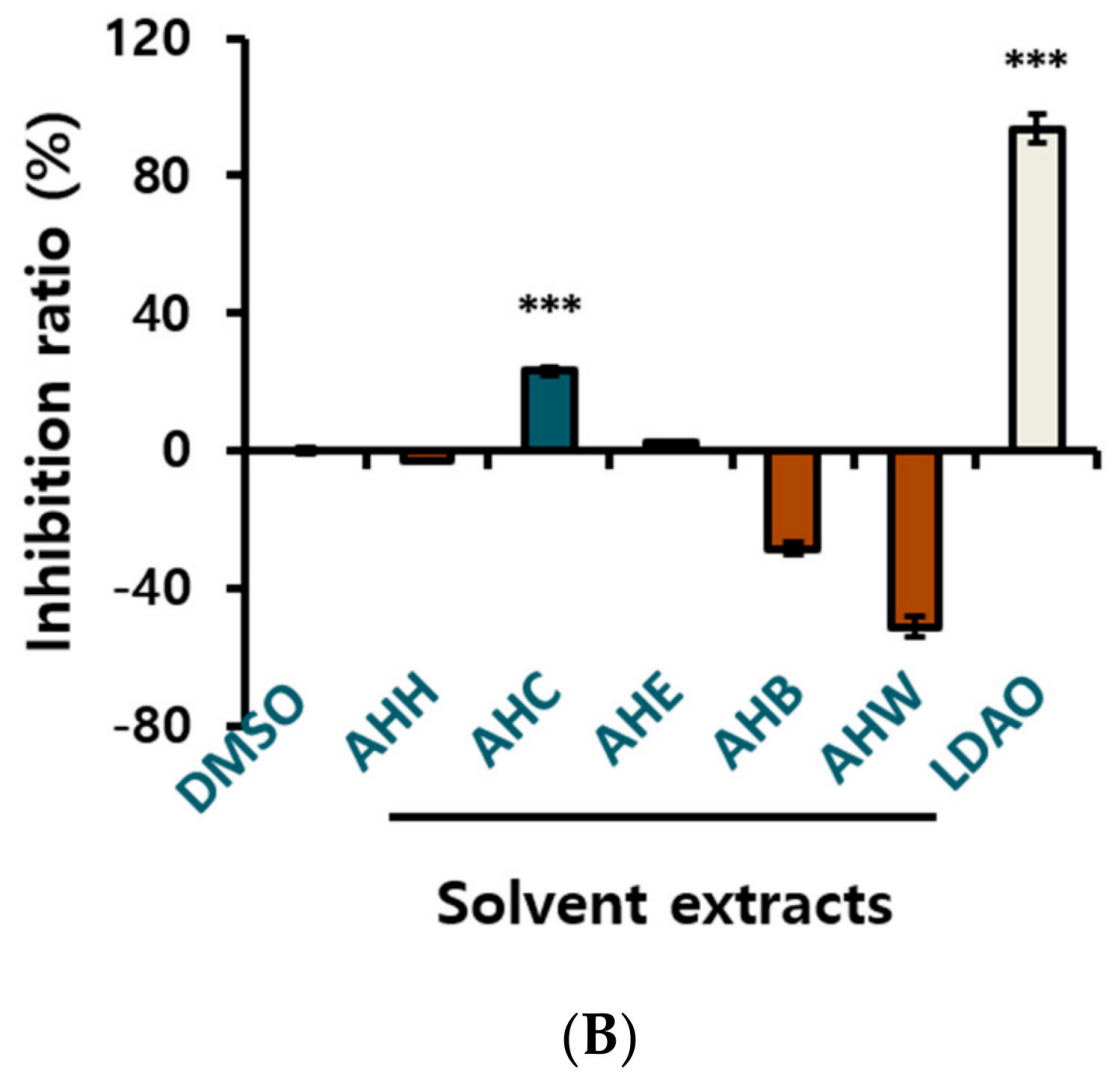
Extraction and isolation from *Alnus hirsuta* by activity guided fractionation: Anti-yeast assay on *Saccharomyces cerevisiae* was employed as a guide for the separation. (**A**) Separation procedure; EtOAc extract (EtOAc ext.) (**B**) Growth inhibition ratio of yeast treated with solvent extracts. The yeast culture solutions were treated with the extracts at a concentration of 1.5 mg/mL. The inhibition ratio was calculated by following equation: Inhibition ratio (%) = 100−[(T−T_0_/N− N_0_) × 100]; T: the OD of yeast solution after incubation; T_0_: the OD of yeast solution before incubation; N: the OD of negative control after incubation; N_0_: the OD of negative control before incubation; 0.1% DMSO in culture medium: negative control; lauryldimethylamine oxide (LDAO) of 688.2 µg/mL: positive control; optical density (OD). Data of *** *p* < 0.005 vs. control.

In order to obtain an extract rich in diarylheptanoids, *A. hirsuta* was roughly extracted using ethyl acetate (EtOAc). The crude extract was partitioned into five solvent extracts including CHCl_3_ extract (AHC, Figure 1A). An anti-yeast assay for the solvent extracts was carried out. Further chromatographic separation of the AHC extracts showing positive yeast-suppressive activity (Figure 1B) was performed to obtain five phytochemicals.

**Figure 2:**
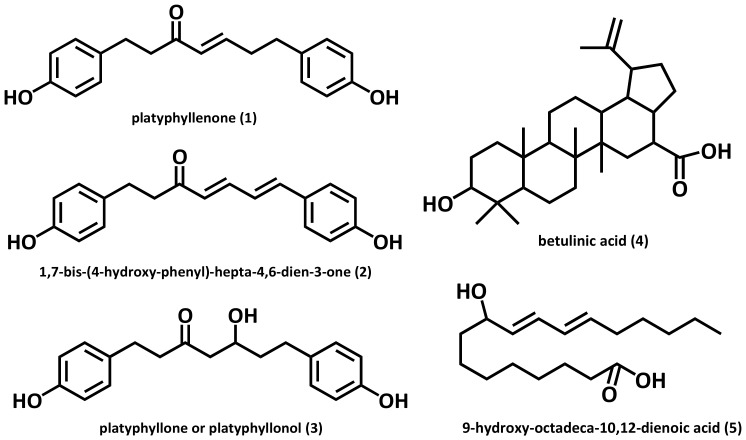
Structures of the compounds **1**–**5** isolated from *A. hirsuta* CHCl_3_ extract (AHC).

The compounds **1**–**5** isolated from the AHC extract were identified as three diarylheptanoids (**1**–**3**: 103.0 mg, 5.0 mg, and 101.0 mg), a triterpenoid (**4**: 7.0 mg), and a conjugated linoleic acid (CLA) (**5**: 25.0 mg). The chemical structures of the compounds (Figure 2) were elucidated by assignment of spectroscopic signals based on 1D and 2D NMR, HRESI-MS, and UV analyses [34,35,36,37,38].

Physiological activities, including the anticancer activity of diarylheptanoids, has been noted over the last decades [24,39]. Diarylheptanoids belong to the polyphenols, a group of plant secondary metabolites with multiple biological properties. Many of them display antioxidative, cytotoxic, or anticancer actions and are increasingly recognized as potential therapeutic agents [40].

Diarylheptanoids are mainly distributed in the roots, rhizomes and barks of *Alpinia, Zingiber, Curcuma* and *Alnus* species. They have come under the spotlight for their anticancer, anti-emetic, estrogenic, antimicrobial, and antioxidant activity [41]. Linear diarylheptanoids from *Alnus glutinosa* bark exhibited strong anticancer activity, considerably higher than that of curcumin, which served as a positive control, in human non-small cell lung carcinoma cell lines [24].

The three compounds **1**–**3** what we separated were identified as linear diarylheptanoids. NMR data of the three diarylheptanoids from A. hirsuta is presented in Table 1. To the best of our knowledge, the two compounds **2** and **5** were isolated from the genus Alnus for the first time. Anti-yeast assay was additionally performed for the five compounds **1**–**5**.

### 2.2. Yeast Inhibitory Activity of the AHC Extract and the Compounds

**Figure 3:**
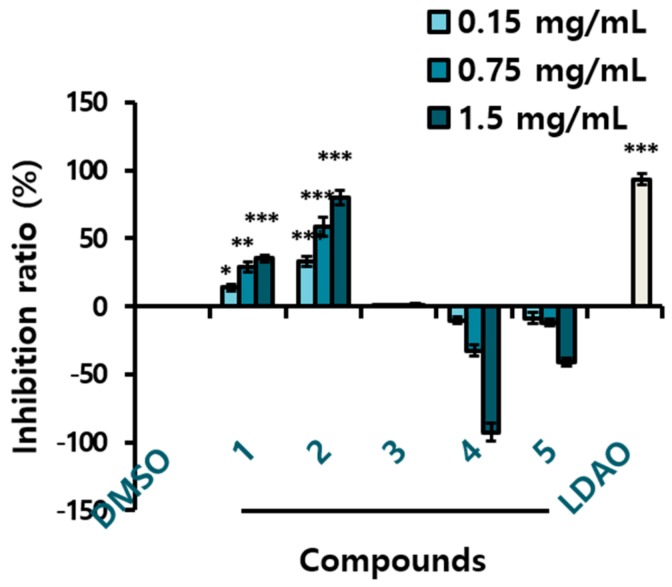
Growth inhibition ratio of yeast treated with the compounds **1**–**5** isolated from the AHC extract. The inhibition ratio was calculated by following equation: Inhibition ratio (%) = 100 − [(T − T_0_/N − N_0_) × 100]; T: the OD of yeast solution after incubation; T_0_: the OD of yeast solution before incubation; N: the OD of negative control after incubation; N_0_: the OD of negative control before incubation; 0.1% DMSO in culture medium: negative control; LDAO of 688.2 µg/mL: positive control; optical density (OD). Data of * *p* < 0.5, ** *p* < 0.05, and *** *p* < 0.005 vs. control.

Yeast is known to have an apoptosis-like programmed cell death process that shares several characteristics with mammalian apoptosis [42]. The MAPK pathways are intracellular signaling modules that exist in all eukaryotes. The yeast Hog1p is a orthologue functionally related to mammalian p38 and JNK [43,44]. Yeast inhibitory assays can be a comprehensive screening method to discover anticancer drugs. We used the yeast growth inhibitory activity as a guideline for fractional separation of the active compounds. The AHC extract presented yeast suppressive activity of 23.2 ± 1.0% (Figure 1B), and three diarylheptanoids (**1**–**3**) displayed positive yeast inhibitory activity of 35.4 ± 2.4%, 80.0 ± 5.0%, and 1.2 ± 0.7%, respectively at the concentration of 1.5 mg/mL (Figure 3). Based on the result, cytotoxic effect by MTT assay was evaluated for the solvent extracts and the compounds **1** and **3** against human cancer cells.

### 2.3. Cytotoxic Effects of the AHC Extract and the Compounds Against Cancer Cells

**Figure 4:**
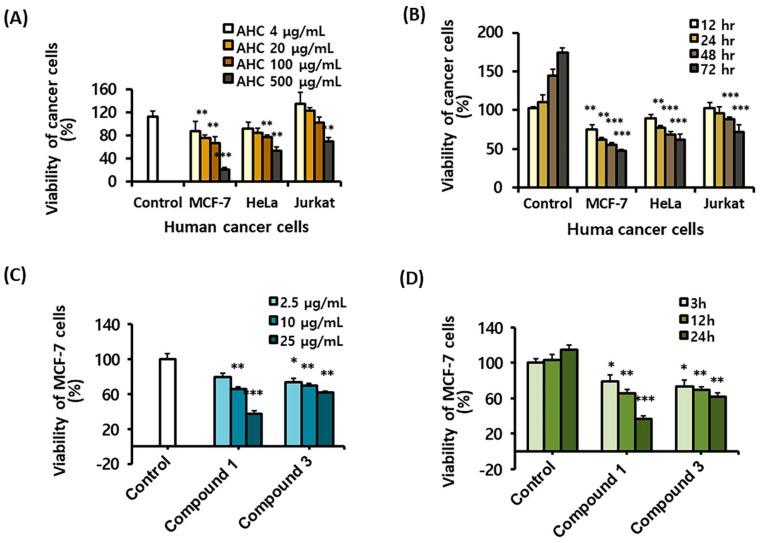
Cytotoxic effects of the AHC extract and the compounds **1** and **3** against various human cancer cell lines. (**A**) Dose dependent effect of AHC after 24 h treatment. (**B**) Time dependent effect of AHC (100 µg/mL). (**C**) Dose dependent effect of compounds **1** and **3** against MCF-7 cells after 24 h treatment. (**D**) Time dependent effect of compounds **1** and **3** (25 µg/mL) against MCF-7 cells. Control: 0.1% DMSO in culture medium. Data of * *p* < 0.5, ** *p* < 0.05, and *** *p* < 0.005 vs. control.

To determine the cytotoxic effects of the extracts and the compounds, an MTT assay was carried out against various human cancer cell lines: Jurkat cells, MCF-7 cells and HeLa cells. The assay was performed preferentially using the five solvent extracts. The AHC extract displayed higher cytotoxic activity against MCF-7 cells than HeLa and Jurkat cells (Figure 4A,B). In the present study, we observed that AHC expressed the cytotoxic effect (IC_50_: 260 μg/mL) against human breast cancer MCF-7 cells in a dose and time-dependent manner (Figure 4A,B). The two diarylheptanoids **1** and **3** displayed significant anti-proliferative activity (IC_50_: 18.1 and 46.9 μg/mL, respectively) against the MCF-7 cells in the analysis (Figure 4C,D).

The IC_50_ values of AHC and compounds were calculated by linear approximation regression of the percentage survival rate versus the treated concentration of substances, which was from the calibration curve measured by absorbance values at 570 nm (Figure 4A,C). It was prospective that AHC containing the two diarylheptanoids would have the potential anticancer activity. To establish the antitumor activity, the biological assays for AHC were advanced.

### 2.4. Effect of the AHC Extract on JNK and p38 Pathways

Enzyme-linked immunosorbent assay (ELISA) is a technique designed for detecting and quantifying substances such as peptides and proteins. ELISA systems can also be used to detect non-immune proteins, not only immune substances. ELISA engaging at least one antibody with an exclusive counterpart antigen is an approach to yield high percent of sensitivity and specificity. ELISA can detect a very small amount of the antibody [45]. ELISA involves the use of enzymes and the specific binding of antigen- antibody. The target proteins to be quantified in ELISA, which has antibodies to form a complex with the proteins, can be detected in liquid samples. According to how it works, ELISA can be divided into four major types: direct, indirect, sandwich, and competitive [46].

Recently, a sandwich enzyme-linked immunosorbent assay (ELISA) was developed by Stynen et al. [47]. A sandwich ELISA using three types of antibodies, capture antibodies, detection antibodies, and HRP-linked secondary antibodies, exhibit in particular highly sensitivity, specificity and excellent quantitative characteristics [48]. The sample with an unknown amount of antigen is immobilized on a well surface of the plate either non-specific adsorption or specific capture by another antibody [49].

The technique requires, in essence, all ligation reagents to bind specifically with detection reagents that can be immobilized to a solid phase and generate a signal that can be properly quantified using enzymes. By washing, only the ligand and its specific binding complements remain ‘immunosorbed by antigen-antibody interactions’ with the solid phase, while unbound nonspecific elements are cleaned away. This antigen-antibody complex is linked to an enzyme-bound secondary antibody. In the final step a substance is added that the enzyme can convert to some detectable signal [50]. The sandwich-type ELISA is considered as a critical tool with highly sensitivity, specificity, and robustness in a report [47,48,51]

For the purpose of exploring the correlation between stress index, inflammation, and anticancer activity, levels of the stress-activated MAPKs of active form were measured parallel with the NF-κB related parameters: Phospho-JNK, phospho-p38 MAPK, phospho-STAT 3, NF-κB, phospho-NF-κB, and phospho-IκB-α. The proteins were detected by sandwich enzyme-linked immunosorbent assay (ELISA) and quantified by the absorbance values at 450 nm. The absorbance values of the reactants reflected the varying degrees of the proteins stimulated by various concentrations of AHC.

**Figure 5:**
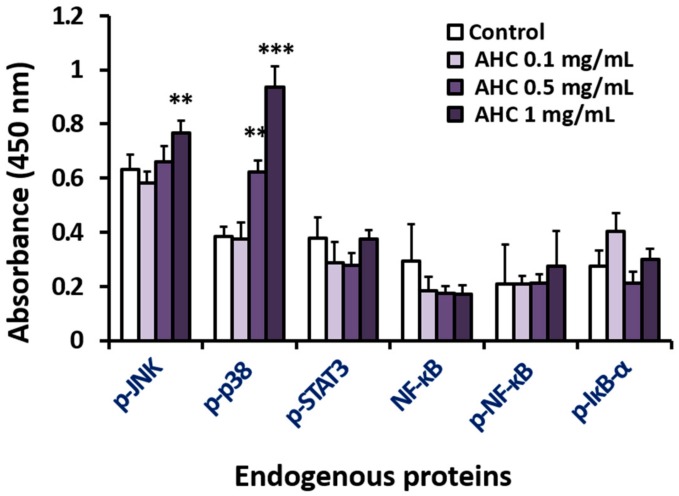
Effects of the AHC extract on the stress activated MAPKs and inflammatory markers in MCF-7 cells. Levels of endogenous proteins after 24 h treatment were detected by enzyme-linked immunosorbent assay (ELISA). phospho-p38 (p-p38), phospho-JNK (p-JNK). Control: 0.1% DMSO in culture medium. Data of **p < 0.05 and ***p < 0.005 vs. control.

The control value of phospho-JNK was higher than other factors, and the level of phospho-JNK in the AHC 1 mg/mL treatment group (0.77 ± 0.04) was 1.2-fold of control group (0.63 ± 0.05). This indicates that the expression of phospho-JNK protein was higher than those of other factors in MCF-7 cells and JNK was more activated by treatment with AHC. The control level of phospho-p38 was comparable to those of the other proteins. However, phospho-p38 in the AHC 1 mg/mL treatment group (0.94 ± 0.08) was 2.5-fold higher than that of control group (0.38 ± 0.04). This data indicates that AHC induced the more activation of p38 than other proteins (Figure 5).

The raised levels of phosphorylated p38 and JNK indicate the activation of the proteins by AHC treatment in MCF-7 cells. The result is consistent with the findings of the papers that showed the increased expressions of JNK and p38 proteins in breast cancer tissues [11,12,13]. Further, p38 seemed to be more activated than JNK and other regulators. However, the levels of inflammatory markers including phospho-STAT3, phospho-NF-κB did not show substantial changes upon AHC treatment.

### 2.5. Effect of the AHC Extract on ROS Production

**Figure 6:**
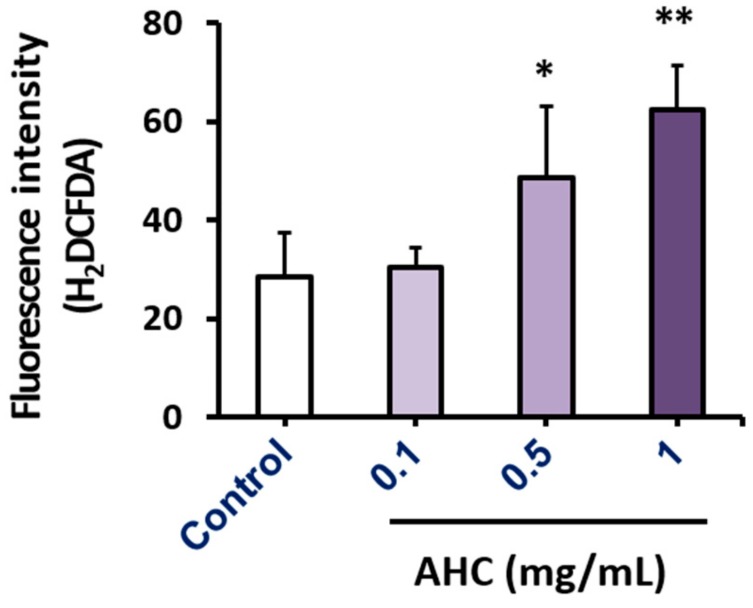
Effects of the AHC extract on ROS generation in MCF-7 cells. Level of ROS generation by AHC after 24 h treatment was monitored by flow cytometry. Control: 0.1% DMSO in culture medium. Data of * *p* < 0.5 and ** *p* < 0.05 vs. control.

Reactive oxygen species (ROS), a group of highly reactive chemicals containing oxygen, are generated in a variety of stress environments. In general, high level of ROS production is considered deleterious to cells and to cause various disorders including senescence, degenerative diseases and cancers. However, ROS can evoke apoptosis processes by playing a role as intracellular messengers for specific growth factors or cytokines. Furthermore, ROS can act as cancer suppressors in some cancer types due to varying antioxidant capacities of the cells [52]. Intracellular ROS generation in MCF-7 cells treated with AHC was measured using ROS indicator H_2_DCFDA. The substance becomes deacetylated or oxidized in the presence of ROS such as hydrogen peroxide, peroxynitrite or hydroxyl radical to produce 2′,7′-dichlorofluorescein (DCF) of green fluorescence. The fluorescence intensity of AHC 1 mg/mL treated group (62.5 ± 8.9) monitored by flow cytometry was higher than two times than the control group (28.6 ± 8.8) (Figure 6). The magnitude of absorbance for the developed color is proportional to the quantity of bound target protein. The elevated level of green fluorescence indicator represents that AHC treatment substantially promoted the ROS generation in MCF-7 cells.

### 2.6. Apoptotic Activity of the AHC Extract

**Figure 7:**
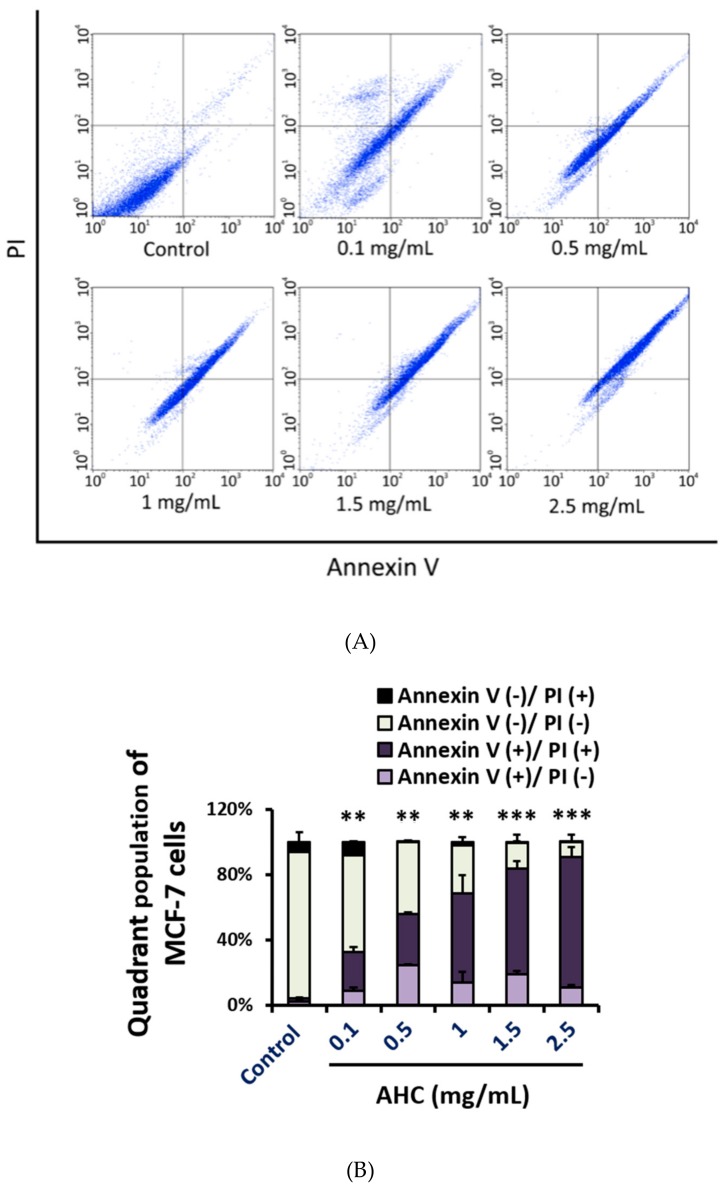
Apoptotic effect of the AHC extract against MCF-7 cells after 24 h treatment was monitored by flow cytometry. (**A**) Quadrant populations of MCF-7 cells (**B**) Diagram of quadrant distribution of the cells. Control: 0.1% DMSO in culture medium. Data of ** *p* < 0.05 and *** *p* < 0.005 vs. late apoptotic cells of control. Propidium iodide (PI).

To establish the in vitro anticancer effect of the AHC extract, we assessed the apoptotic effect against MCF-7 cells by flow cytometry analysis using Annexin V and PI double-staining dyes. The assay was based on the capacity of Annexin V to bind to the exposed phosphatidylserine (PS) of the apoptotic cells with high affinity. Propidium iodide (PI) binding to DNA of the cells was employed to distinguish the late apoptotic and necrotic cells. The quadrant populations of the cells represent the viable [Annexin V(−)/PI(−)], early apoptotic [Annexin V(+)/PI(−)], late apoptotic [Annexin V(+)/PI(+)], and necrotic [Annexin V(−)/PI(+)] cells, respectively. The number of viable cells of AHC 2.5 mg/mL treated group was significantly reduced (9.2 ± 4.7%) compared to that of control group (89.3 ± 6%). The percentages of early apoptotic cells and late apoptotic cells of the AHC 2.5 mg/mL group were 10.8 ± 1.6% and 80.0 ± 6.3%, which were significantly higher than the control groups with 2.5 ± 0.5% and 2.0 ± 0.2%, respectively (Figure 7).

The quadrant data following 24 h treatment reveal that AHC 2.5 mg/mL elicited the MCF-7 cells into early and late apoptotic states in a concentration dependent manner. Further, the result demonstrates that AHC leads MCF-7 cells primarily to late apoptotic stage. However, the percentage of necrosis by AHC treatment was not high compared to that of control group.

### 2.7. Cell Cycle Arrest Effect by the AHC Extract

**Figure 8:**
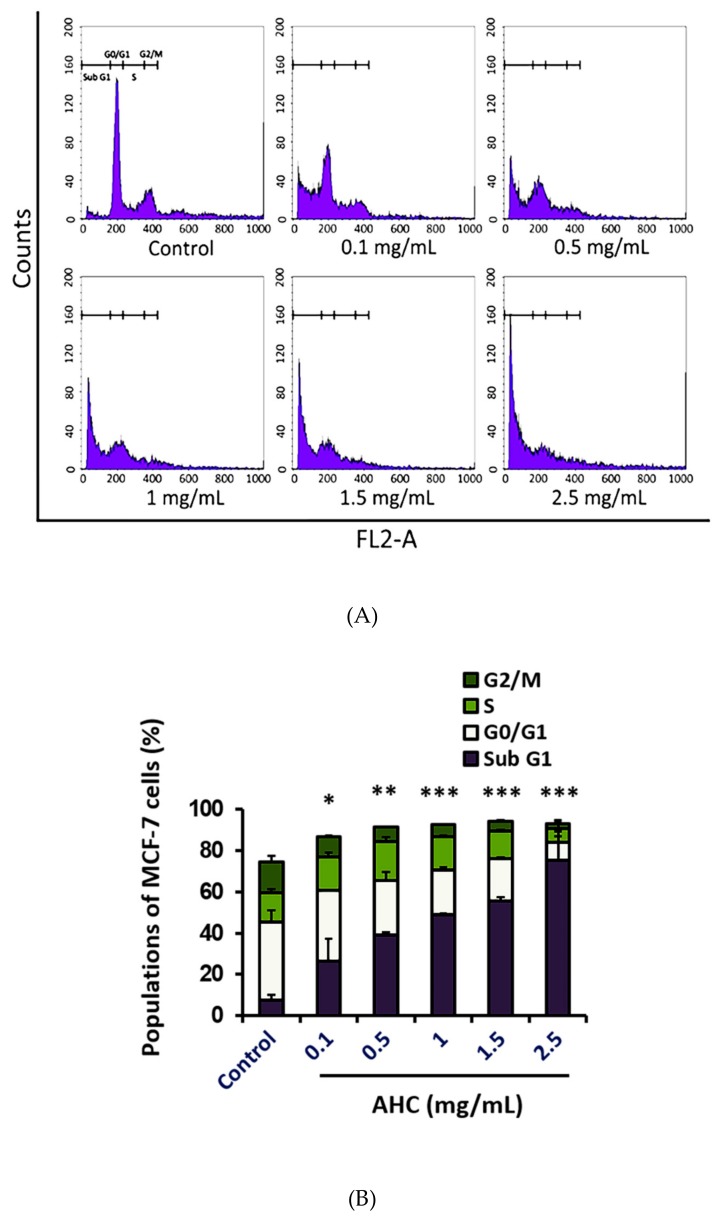
Cell cycle arrest effect of the AHC extract against MCF-7 cells. (**A**) Histograms of cell cycle distribution (**B**) Diagram of each cell phase distribution. Histogram of DNA contents after 24 h treatment monitored by flow cytometry was presented. Control: 0.1% DMSO in culture medium. Data of * *p* < 0.5, ** *p* < 0.05, and *** *p* < 0.005 vs. Sub G1 phase of control.

The regulation of cell cycle is critical for the normal proliferation development of multicellular organisms [53]. To determine the antiproliferative activity of AHC, we analyzed the DNA contents of each cell cycle population of MCF-7 cells by flow cytometry using propidium iodide (PI). The populations of the cells in Sub G1, G0/G1, S and G2/M phases were identified by monitoring a fluorescent probe binding to DNA of the cells. The data indicate that 75.5 ± 13.7% of cells treated with AHC 2.5 mg/mL was distributed in Sub G1 phase, compared to 7.7 ± 2.5% of control cells (Figure 8).

The fixed cells with lower DNA contents, the DNA-damaged cells including apoptotic or necrotic cells, can be distributed in the ‘Sub G1′ phase when monitored [54]. Irreparable DNA damage may force the cancer cells to withdraw from the cell cycle so that they cannot replicate chromosomes. DNA damage prevents the cells from entering mitosis, the proliferative phase [55]. Increasing the concentration of AHC caused a raised population of MCF-7 cells in the Sub G1 phase, which was accompanied by a proportional decrease of the cells in the G0/G1-S-G2/M phases. Treatment of high concentrations of AHC for 24 h shifted the majority of the cells into Sub G1 phase compared to the control cells, which indicates the apoptosis induced by AHC. Our study displayed that inducing the cycle arrest in MCF-7 cells was an important basis of anti-proliferative activity of the AHC extract as well.

## 3. Discussion

Significant advances in our understanding for cancer in the last century gave led to improved treatments and prevention of the disease, however, cancer still poses a health issue worldwide [2]. There have been steadily increased interests in the treatment with alternative therapies by natural products in the field of cancer research. Since the 1980s, a number of new compounds with indications for cancer treatment have been approved for commercialization, about half of which were derived from or based on the natural products. Clinical evidences for traditional utility of natural products with the relatively easy access contribute to active researches on various diseases, including cancer. Therefore, most of anticancer compounds were discovered from natural products, most notably plants [56]. Nine plant-derived compounds have been approved as anticancer drugs in the USA up to 2011 [57]. Nature holds the substances of plant and marine origin that have anticancer properties including taxanes, camptothecin, and halichondrin [58].

With the development of molecular targets based on the proteins, there is an increasing demand for elucidation of the molecular pathway for the treatment. Natural products will play an essential role in satisfying the demand through the continuing investigation of the molecular signaling pathway. Our study was purposed to evaluate the effect of *Alnus hirsuta* and to elucidating the mechanistic pathway of anticancer activity against the cancer cells.

We preferentially partitioned the crude extract into five solvent extracts including CHCl_3_ extract (AHC) and obtained five compounds **1**–**5** from AHC (Figure 1A) using anti-yeast assay as a guideline for separation (Figure 1B). The amounts of the isolated compounds **1**–**5** were 103.0 mg, 5.0 mg, 101.0 mg, 7.0 mg, and 25.0 mg, respectively. We carried out spectroscopic analyses such as NMR, mass, UV for the structure elucidation of the compounds **1**–**5** (Figure 2).

Compound **2** showed the highest yeast suppressive effect in the anti-yeast assay performed for the five compounds (Figure 3). Most of compound **2**, the minor constituent, was exhausted by carrying out the anti-yeast test and structural analysis. Therefore, no further experiments with compound **2** could be conducted. According to the hypothesis, we infer that compound **2** would have shown the highest antiproliferative activity. Few studies have been reported on compound **2**, a phytochemical that is not commonly found in plants. We yet speculate that compound **2**, the phenolic chemical possessing two unsaturated moieties in the structure can have a potential various biological activity, aside from our hypothesis.

Compound **3** showed a very slight yeast inhibitory effect, but we determined it to be positive in that the yeast did not at least proliferate. In addition, we expected some physiological activity, since compound **3** is a diarylheptanoid, identically with compounds **1** and **2**. During further experiments, only cytotoxicity assay could be carried out with compounds **1** and **3** on account of the limited amounts.

In order to explore the activity of extracts and the compounds on cell types, we selected the three types of cancer cells from different origins, a type of hematological malignant cells and two types of solid cancer cells. The five solvent extracts including AHC were treated on the three human cancer cells, respectively: T cell lymphoma Jurkat cells, ER positive breast cancer MCF-7 cells [59] and ER-negative cervical cancer HeLa cells [60]. The cancer cells exhibited different degrees of inhibition by the five extracts. Among them, the AHC extract showed an antiproliferative activity against two types of carcinoma among the three different cancer cells, especially high activity against MCF-7 cells (Figure 4A,B). Compounds **1** and **3**, the two diarylheptanoids, presented significant cytotoxicity against the cells (Figure 4C,D). Consistently, the compounds **1** and **3** were previously reported to have cytotoxic acting on lung cancer cells [61].

For Figure 4A,C comparing viability versus concentrations, a 0.1% DMSO treated single control was used, since the control did not need to be used in multiple concentrations even though multiple concentrations of the extract or compounds were adopted. For Figure 4B,D comparing viability versus time, the survival rates over time of groups treated with AHC 100 μg/mL or compounds 25 μg/mL were compared with those of the control group. Therefore, a respective control group for each time was used.

To establish the activity of AHC estimated to have potential anticancer activity, the biological assays using the extract were advanced. We investigated the effect of the AHC extract on the activation of p38 and JNK, with the suppressive effect of breast cancer MCF-7 cells. The anticancer activity and the mechanism were assessed by cell viability, apoptotic effect, DNA contents, the levels of phospho-JNK and phospho-p38, and ROS generation.

Apoptosis is essential for the development and survival of multicellular organisms and is a highly regulated and sophisticated process triggered by various factors. Redox-dependent mechanism of release of cytochrome c from mitochondria to cytosol is regarded as a central event in apoptotic initiation. Reversible activation of caspases is reportedly important mechanism in the apoptotic execution. Besides, GSH/GSSG redox system plays an essential role in cellular redox regulation and apoptosis [22]. Dysregulated apoptosis may often lead to various pathologic conditions. Characteristic of malignant tumors including breast cancer are manifested by the reduced apoptosis compared to normal cells. Oncogenic changes by are often caused by overexpression of anti-apoptotic genes and/or downregulation of pro-apoptotic genes. These genetic aberrations are mediated by functional loss or mutations of p53, a nuclear transcription factor with a pro-apoptotic function [62].

The ROS generation can affect cancer cells, resulting in the apoptotic cell death and cell cycle arrest via the activation of MAPKs. To detoxify the ROS generated from tumor cells, the cells express increased levels of antioxidant proteins, suggesting that a delicate balance of intracellular ROS levels is required for the function of cancer cells. In order to operate the function to generate the proteins effectively via mediated the signal pathways, cellular ROS-sensing stress regulators are required [63]. In addition, activation of the immune function of normal cells by the antioxidant activity can contribute to the suppression of tumor cells. A number of chemotherapeutic strategies have been designed to induce the apoptosis by increasing the ROS level with a goal of damaging the tumor cells [21].

Apoptosis elicited by ROS generation accompanies complex biochemical events such as mitotic arrest and activation of JNK and p38. The effect is mediated through a p53-dependent regulation of apoptotic relevant molecules [7,62]. The MAPKs are activated by phosphorylation in response to numerous external factors. JNK and p38 are weakly activated by growth factors but respond strongly to stress signals including tumor necrosis factor (TNF), oxidative damage, and chemotherapeutic drugs.

Western blotting (WB, also called immunoblotting) and ELISAs are techniques conventionally used to detect target proteins. WB and ELISA resemble acceptably analogous as the two experiments are alternatives each other in some cases. However, even though the two approaches are both based on immunodetection, their application is not exact identical.

It is regarded that a crucial factor determining which technique is more appropriate for the experiment depends on the purpose of the study. WB is a useful technique with high specificity that identifies the target proteins through their molecular weight (MW). Using western blotting enables qualitative analysis inclusive of confirmation the homogeneity, presence of additional bands and truncated products of proteins. The quantification of WB reflects the relative amounts as a ratio of each protein band relative to the control [64]. ELISA is considered as an approach that exhibits high sensitivity and specificity comparable to Western blotting [50]. ELISA is often used to quantitate a specific protein which exists in a mixture of various proteins. Therefore, it can be more reasonable that sandwich ELISA showing excellent quantitative characteristics such as reproducibility [48] is chosen for monitoring protein samples of varying concentrations.

The purpose of our study was to investigate the quantitative changes of endogenous proteins including JNK and p38 in MCF-7 cells by AHC treatment to reveal their activation degrees and to determine the apoptotic pathway based on the extent. It is consequently considered that ELISA is more valid for the accurate quantitation of the quantitative change, even though the application of WB is conceivable to our case.

ELISA is a very mature method for the detection of various targets. Advantages of ELISA are that the process is relatively straightforward and does not require lots of time to carry out, so it is often used for both diagnostic and research purposes [46]. However, these benefits contain a risk of making ELISA appear to be a lightweight technique. But ELISA is rather a trustworthy assay, as evidenced by innumerable literatures. The assay is highly sensitive, specific and shows excellent quantitative characteristics such as reproducibility, dilution linearity and recovery (over 97%) [48,51,65,66]. The ELISA kit does not support the total protein levels. However, since the amount of total protein does not change with treatment of chemicals, it can be inferred that an increment of phosphorylated protein indicates an increased activation of the protein [67].

We measured the levels of phospho-JNK and phospho-p38 by sandwich enzyme-linked immunosorbent assay (ELISA). Our result of ELISA showed that the expression level of phospho-JNK protein in MCF-7 cells was higher than other factors. The phospho-JNK elevated by treatment with AHC. The expression of phospho-p38 in the AHC treated group was significantly higher than that of control group. The data of Figure 5 indicate that AHC activated p38 the most, compared to other modulators. The function of the stress activated protein kinase (SAPK) is often proposed as a tumor suppressor [68]. The level of ROS generation and the activation of p38 and JNK in the AHC-treated cells were higher than the control. The data indicate that AHC produced ROS and provoked the signal of p38 and JNK in MCF-7 cells (Figure 5 and Figure 6).

Quadrant data of flow cytometry displayed that AHC treatment induced the MCF-7 cells into apoptotic states (Figure 7). The data are the result of excluding the disrupted cells, since analysis of apoptosis and cell cycle by flow cytometry is practicable using only intact cells, not debris. Therefore, higher concentrations of AHC were applied to flow cytometry than MTT assay. In practice the AHC extract may possess potentially higher cytotoxicity against MCF-7 cells than the monitored results.

The activation of JNK and p38 is associated with apoptotic cell death. The dysregulation of p38 levels tend to advance the stages and shorten the survival in breast cancer patients. Albeit the roles of JNK and p38 in breast cancer are still ambiguous, studies indicated a role of the p38 pathway in tumor suppression. The increased level of p38 activation is related with apoptosis, and the p38 can be activated by anticancer agents [7,16,69,70]. Loss of JNK signaling can cause genomic instability and the development of breast cancer. The deficiency of JNK or increased expression of the inactivated protein can enhance tumor growth or induces drug resistance in various cancer cell lines. In addition, an association between reduced JNK signal transduction and tumor development was reported [71]. The tumor suppressive functions of JNK are often regarded to be related to its pro-apoptotic activity [7,11,71]. We suggest that cytotoxic activity of the AHC extract against MCF-7 cells might be induced by apoptosis mediated by p38 and JNK (Figure 5 and Figure 7). These effects can be supported by the using of MAP kinase inhibitors such as SB203580 (p38 MAPK inhibitor) [72].

ROS generated in cells exists in equilibrium with a variety of antioxidant defenses. ROS below medium doses are important for regulation of normal physiological functions involved in development such as cell cycle progression and proliferation, differentiation, migration and cell death. ROS also play an important role in the immune system, maintenance of the redox balance and have been implicated in activation of various cellular signaling pathways. High cellular levels of ROS cause damage to proteins, nucleic acids, lipids, membranes and organelles, which can lead to activation of cell death processes such as apoptosis [73].

With respect to apoptosis, ROS is in general associated with induction of death. In this case, ROS appears to be involved in both apoptosis by cytochrome c release at the mitochondrial level and death receptor-mediated apoptosis. Modulation of intracellular ROS levels by drugs has the potential to normalize apoptosis of regulatory abnormalities in cells. The functional importance of ROS generation for the activation of death mechanisms has been demonstrated by inhibitor studies such as antioxidant [74]. Therefore, the delicate balance between the activation of antioxidant devices and apoptosis induced by ROS seems to be vital for cancer treatment to activate the immune function including antioxidant system or to lead to cancer cells death. Our data demonstrate that AHC produced ROS and induced apoptosis of MCF-7 cells (Figure 6 and Figure 7). The findings display that the ROS generation could be a strategy for the treatment of some types of breast cancer.

MAPK signaling related to DNA damage response is known to potentially influence tumor cells [75]. The p38 regulates both the G2/M as well as a G1/S cell cycle checkpoint in response to cellular stress such as DNA damage [17]. Monitored DNA contents indicated that the AHC extract led the cycle arrest of MCF-7 cells. After 24 h treatment with various concentrations of AHC, a significant increase in the number of cells accumulated at Sub G1 phase was observed. Most of the tumor cells treated with AHC residing on Sub G1 phase is considered apoptotic cells or DNA-damaged cells, rather than the necrotic cells (Figure 7 and Figure 8). It is conceivable that AHC regulate the cell cycle and slow down the replication of the cells by modulating MAPK signal transduction (Figure 5). As a result, our results show that AHC treatment resulted in the cell cycle arrest of MCF-7 cells.

**Figure 9:**
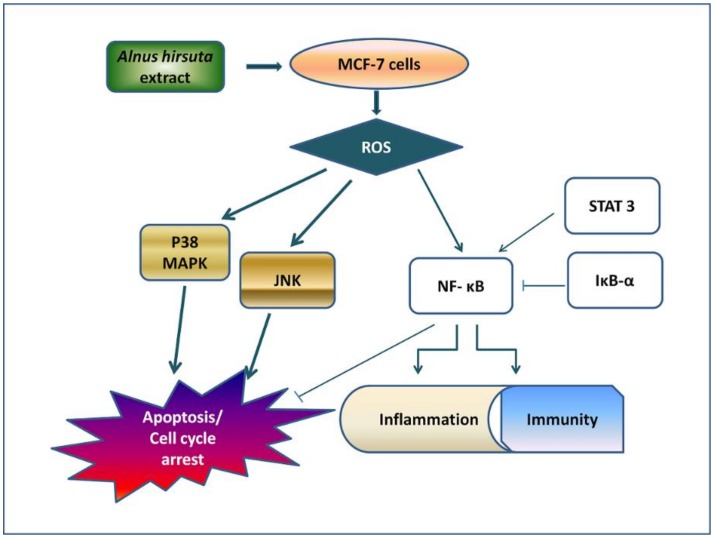
Schematic illustration of the potential regulatory pathway of the AHC extract against MCF-7 cells.

The AHC extract and two diarylheptanoids significantly reduced the survival rate of MCF-7 cells. Taken together, the effects might be exerted by the AHC extract affecting the factors of the above signal transduction pathways. Based on these data, we summarized the potential regulatory route of the AHC extract in MCF-7 cells as depicted in Figure 9. Current study presents that activation of the p38 and JNK can lead apoptotic effect in MCF-7 cells. The pathway is consistent with the route suggested by previous researches [21,53,75,76,77,78,79,80,81].

Numerous natural products show anticancer activity by the cooperation and/or downstream the signals transduction of p38 and JNK, accompanying the apoptotic effect. Our current data represent that the *A. hirsuta* extract possesses such effects as well. No study previously has reported the anticancer activity of *A. hirsuta* in MCF-7 cells. These results provide an information that *A. hirsuta* may activate the apoptotic pathway in MCF-7 cells by induction of phosphorylation of JNK and p38 and ROS generation. We believe that the properties of *A. hirsuta* may contribute to the research for the development of effective anticancer agents for the treatment of some types of human breast cancer.

However, our approach has some limitations. First, most of biological experiments except cytotoxicity assay were mainly advanced with extract not with the compounds. To acquire a single substance isolated takes in general about five to six months. In fact, obtaining a few milligram quantities of the isolated single compound for us took about six months. The hardship of further separation of the compounds made it difficult to work additional experiments with them. Compound 2, the minor component, in particular could not be used in any further experiments including cytotoxicity assays on account of the insufficient quantity available, despite showing the highest yeast inhibitory effect. Second, we could not assess the effect of AHC on ERK, one of the important MAPKs families, since the microwells of the ELISA kit what we used did not contain the ERK antibody. The third is that we did not experiment with diverse breast cancer cell lines. In order to more accurately assess the effects of AHC, there are still many challenges for us. Further experiment is demanded on more diverse cell lines, including triple negative (ER-, PR- and HER2-) breast cancer cell lines. To assess the safety of *A. hirsuta*, experimentation with non-carcinoma cells should be added.

ER, PR, HER2, and EGFR are the receptors presented in MCF-7 cells [5]. The characteristics of MCF-7 cells provide the basis for a good model for breast cancer research. We experimented with only one type of breast cancer MCF-7 cells, but this may provide clues to the strategies for the treatment of breast cancer. Although higher IC_50_ values were shown, HeLa and Jurkat cells also presented cytotoxicity in MTT assay. A document on lung cancer cells support the anticancer activity of the two diarylheptanoids that we isolated from AHC [61]. Therefore, we can estimate the likelihood of activity as described above against these cells. However, it is unclear that the anticancer mechanism of AHC can be applied to all types of carcinoma or all types of breast cancer.

The evaluation of effect of the AHC extract on ERK, another important member of MAPKs, is one of the themes for further study. Further works using kinase inhibitors or western blotting could be done to clarify the relevance of activation of p38 and JNK to the increased apoptosis of MCF-7 cells. Antioxidants are the crucial tools to determine whether AHC is involved in direct in the mechanism of ROS generation related with activation of p38 and JNK and apoptotic event. The experiment with the antioxidants is also one of the subjects that we should achieve.

Current study offers a clue that *A. hirsuta*, containing diarylheptanoids, has a potential for anticancer activity against human cancer cells. A great deal of assignment was imposed to be desired along with the hope to positively evaluate what we have found, since there are shortcomings in many respects and additional challenges implemented.

## 4. Materials and Methods

### 4.1. Plant Materials: Extraction and Isolation

Dried stem bark of *A. hirsuta* obtained from Jecheon district in Korea was identified by Prof. Hyoung-Tak Im of Chonnam National University (CNU). A voucher specimen (JNU-A017) was deposited at the Herbarium of College of Pharmacy, CNU in Korea. The stem bark of *A. hirsuta* (5.0 kg) was pulverized and then extracted with 5 L ethyl acetate (EtOAc) at room temperature three times for 24 h. The extracted solution was concentrated in vacuo to 608× *g* residue of the crude EtOAc extract. The extract was suspended in H_2_O and partitioned sequentially with 2 L of each solvent of hexane, CHCl_3_, EtOAc, n-BuOH and H_2_O, resulting in a residue of AHH (65 g), AHC (81 g), AHE (260 g), AHB (127 g), and AHW (75 g), respectively. The AHC extract (40 g) was further separated into seven fractions (AHC 1–7) by eluent CHCl_3_-MeOH. Open column chromatography (silica-gel, Kieselgel 60, 1.07734.9025, Merck, Darmstadt, Germany) and Medium Pressure Liquid Chromatography (MPLC, YFLC 530, YAMAZEN, Osaka, Japan) with an ODS-SM-50B column were used for the separation and isolation. Isolated compounds were purified by Sephadex LH-20 (Pharmacia, Piscataway, NJ, USA). Individual compounds were isolated from subfraction AHC 4 of the AHC extract.

The structures of the compounds were elucidated by NMR spectrometry (Unity Inova, Varian, Palo Alto, CA, USA). The spectrometry of high-resolution mass and ultraviolet (UV) was employed for verifying the chemicals. 15T FT-ICR was engaged to measure the mass of the compounds in high resolution. A V-530 UV/VIS spectrophotometer (Jasco, Tokyo, Japan) was used to obtain UV spectra. A fractionation scheme was shown in Figure 1A. Aliquots of the solvent extracts and the isolated compounds were dissolved in dimethyl sulfoxide (DMSO) and diluted in desired concentrations with phosphate buffered saline (PBS) for biological assays.

### 4.2. Evaluation of Yeast Suppression Effects of the Extracts and Compounds

As a fundamental screening test for the cytotoxic activity of substances, we performed a yeast growth suppression assay using a strain, *Saccharomyces cerevisiae* variant (KCTC 17796) (Korean Collection for type culture, KCTC, Daejeon, Korea). The yeast was incubated for 24 h in a temperature-controlled shaker at 25 °C and 200 rpm. The optical density (OD) of the yeast culture was measured at 600 nm using a cell density meter-Ultrospec^TM^ 10 (Amersham Biosciences, Piscataway, NJ, USA).

### 4.3. Cytotoxicity Determination of the Extracts and the Compounds

To evaluate the cytotoxicity of the extracts and the compounds against human cancer cell lines, MTT assay was carried out on MCF-7, HeLa, and Jurkat cells. Cells were purchased from the Korean Cell Line Bank (KCLB). The cells were cultured in RPMI 1640 medium (Gibco, Langley, OK, USA) supplemented with heat-inactivated 10% fetal bovine serum (FBS), 100 μg/mL of penicillin, 100 μg/mL of streptomycin, and 0.25 μg/mL of amphotericin B under a humidified atmosphere with 5% CO_2_ at 37 °C. Viabilities of tumor cells were quantified by the reducing activity of living cells converting the yellow dye 3-(4,5-dimethyl-2-thiazolyl)-2,5-diphenyl-2*H*-terazolium bromide (MTT) to a blue formazan product 1-(4,5-dimethylthiazol-2-yl)-3,5-diphenylformazan. Cell viability was evaluated by the absorbance values measured at 570 nm using a microplate spectrophotometer (Eon, Biotek, Winooski, VT, USA). The viability was calculated according to following formula:

Cell viability (%) = A_T_/A_NT_× 100

A_T_: the absorbance of cells treated with *A. hirsuta* extracts; A_NT_: the absorbance of non-treated cells.

### 4.4. Detection of Endogenous Proteins in the AHC Extract-Treated MCF-7 Cells

To investigate the effect of the AHC extract on p38, JNK, and the inflammation-related factors, we measured the endogenous levels of the proteins: Phospho-JNK, phospho-p38, phospho-STAT 3, NF-κB, phospho-NF-κB, and phospho-IκB-α. The amounts of the endogenous proteins were quantified by indirect sandwich enzyme-linked immunosorbent assay (ELISA). Each well of the kit used in our experiment was coated with capture antibodies of the six proteins listed above.

MCF-7 cells seeded at 5×10^5^ cells/mL in 6-well plate were treated with AHC and incubated at 37 °C for 24 h. Lysis buffer containing phenylmethyl sulfonyl fluoride (PMSF) were added to the harvested cells, which was stored for 2 h on ice with shaking. Scraped cells from the plates were transferred to microtubes. After centrifugation at 4 °C for 10 min, the supernatant (cell lysate) was transferred to a new tube. Diluted samples of AHC-treated cell lysates were put into the each well of the kit coated with capture antibodies of the six proteins listed above. The microwell was incubated overnight at 4 °C following seal. After incubation, the target proteins were seized by the coated capture antibodies.

Following extensive washing, detection antibodies were added to each well. It was incubated at 37 °C for 1 h sealing. The detection antibodies were added to detect the captured target proteins. HRP-Ab^o^ (2nd Ab^o^) was added to each well after cleaning the well. The HRP-linked secondary antibody was used to recognize the bound detection antibody. Antigen-bound detection antibodies were targeted by enzyme-conjugated secondary antibody. The reactants containing the antigen-antibody complexes were incubated at 37 °C for 30 min sealing. 3,3′,5,5′-Tetramethylbenzidine (TMB) substrate was added to each well to develop the color after washing the well. It was incubated at 37 °C for 10 min sealing. Each well with a stop solution was gently shaken for a few seconds. The quantitative levels of the detected factors were assessed by the absorbance values at 450 nm. Absorbance was measured within 30 min after adding stop solution. PathScan^®^ Multi-Target Sandwich ELISA Kit from Cell Signaling Technology (Danvers, MA, USA) was used for this assay.

### 4.5. Measurement of ROS Generation in MCF-7 Cells by the AHC Extract

To assess the activity of AHC producing the oxidative stress, the ROS level of the AHC-treated MCF-7 cells was measured. 2′,7′-Dichlorodihydrofluorescein diacetate (H_2_DCFDA), a derivative of reduced fluorescein, is a cell-permeant indicator of ROS. MCF-7 cells seeded at 5×10^5^ cells/mL in 6-well plate were treated with AHC and incubated at 37 °C for 24 h. The working solution of H_2_DCFDA probe dissolved in DMSO was freshly prepared prior to use. H_2_DCFDA dye 1 mM were loaded to the harvested cells washed with pre-warmed PBS and incubated for 1 h in an incubator of 37 °C, 5% CO_2,_ protecting the cells from light. The culture medium containing H_2_DCFDA were washed with pre-warmed PBS to be removed. Fresh medium was added to the cells and incubated for 1 h, protecting the cells from light. Fluorescence level of ROS was immediately assessed by analyzing the cells in green fluorescence. H_2_DCFDA used in the analysis was purchased from Invitrogen (San Francisco, CA, USA). ROS level was monitored by Cell Quest software of flow cytometry using BD FACSCalibur (BD Biosciences, San Jose, CA, USA).

### 4.6. Evaluation of Apoptotic Effect of the AHC Extract on MCF-7 Cells

Annexin V-FITC/PI assay was performed to assess the apoptotic effect of the extract against MCF-7 cells. MCF-7 cells seeded at 5 × 10^5^ cells/mL in 6-well plate were treated with AHC and incubated at 37 °C for 24 h. The harvested cells washed with cold PBS were centrifuged. The centrifuged cells suspended in binding buffer were transferred to the round-bottom tubes. The cells were stained with 5 µL of Annexin-FITC solution and 10 µL of PI iodide (PI) solution from Annexin V-FITC/PI Apoptosis Detection Kit (Sigma-Aldrich, Darmstadt, Germany) and shaded for at least 10 min. The stained cells were analyzed by flow cytometry, followed by quadrant statistics analysis.

### 4.7. Cell Cycle Analysis of MCF-7 Cells Treated with the AHC Extract

To establish the antiproliferative effect of the extract, DNA contents of the cells were measured. MCF-7 cells seeded at 5 × 10^5^ cells/mL in 6-well plate were treated with AHC and incubated at 37 °C for 24 h. The harvested cells washed with cold PBS were centrifuged. The cells were fixed with 70% ethanol for at least 2 h. The fixed cells washed with cold PBS were centrifuged. 5 μL of RNase A, DNase and protease-free 10 mg/mL (Thermo Fisher, San Francisco, CA, USA) was added to the cells, which was incubated for 30 min at 37 °C. 50 μL of propidium iodide (PI) 1 mg/mL (Sigma-Aldrich) was added to the cells, which was incubated for at least 10 min at room temperature shaded. The contents of DNA stained by PI were quantified by flow cytometry.

### 4.8. Statistical Analysis

Each experiment was performed at least three times. Data are presented as mean ± SD. *p* values were calculated using the Student’s *t*-test by Sigma Plot software, and statistical significance of the data was presented as * *p* < 0.5, ** *p* < 0.05, and *** *p* < 0.005. Data of *p* < 0.05 were accepted as being statistically significant.

## 5. Conclusions

Uncontrolled growth of malignant cells in the mammary epithelial tissue is a characteristic of breast cancer. JNK and p38 are vital MAPKs associated with apoptotic cell death in tumors. The *A*. *hirsuta* containing platyphyllenone (**1**) and platyphyllone (**3**) as main compounds showed an in vitro anticancer effect in MCF-7 cells. We report the biological activities of *A. hirsuta* extract (AHC) associated with the control the apoptosis and JNK and p38 in the cells. The two linear diarylheptanoids **1** and **3** and the AHC extract exhibited cytotoxic effects on the MCF-7 cells in MTT assay. Treatment of the AHC extract induced ROS generation and elevated the endogenous levels of phospho-JNK and phospho-p38 in MCF-7 cells. The p38 was activated by AHC treatment the most, comparing with the other modulators. AHC resulted in apoptosis and cell cycle arrest of the cells. We propose that the in vitro anticancer activity of the *A. hirsuta* extract against MCF 7 cells could be achieved, at least in part, by promotion of cell apoptosis. The fact implies that *A. hirsuta* extract containing a plenty of active materials including diarylheptanoids can provide a prospective for the development of resource as anticancer drugs. We desire that our current research should provide fruitful avenues for future cancer research.

## Figures and Tables

**Table 1:** NMR data of three diarylheptanoids from *Alnus hirsuta.*

Position	Compound 1			Compound 2			Compound 3		
	δ_H_	Multiplicity	δ_C_	δ_H_	Multiplicity	δ_C_	δ_H_	Multiplicity	δ_C_
**1**	2.74 (t, *J* = 7.7 Hz)	CH_2_	30.7	2.83 (t, *J* = 15.5 Hz)	CH_2_	31.0	2.73 (m, *J* = 12.0 Hz)	CH_2_	29.9
**2**	2.78 (t, *J* = 7.7 Hz)	CH_2_	42.8	2.90 (t, *J* = 14.5 Hz)	CH_2_	43.3	2.71 (m, *J* = 11.5 Hz)	CH_2_	46.5
**3**	-	C	203.0	-	C	203.1	-	C	212.1
**4**	6.03 (d, *J* = 16.0 Hz)	CH	131.7	6.26 (d, *J* = 15.5 Hz)	CH	146.4	2.61 (m, H-4)	CH_2_	51.3
**5**	6.84 (dt, *J* = 16.0 Hz)	CH	149.4	7.37 (d, *J* = 13.0 Hz)	CH	143.8	4.0 (*J* = 13.0 Hz)	CH	68.4
**6**	2.42 (t, *J* = 7.0 Hz)	CH_2_	35.8	6.96 (d, *J* = 15.5 Hz)	CH	125.1	1.66 (dd, *J* = 9.0 Hz)	CH_2_	40.0
**7**	2.43 (t, *J* = 7.5 Hz)	CH_2_	34.6	6.84 (t, *J* = 15.5 Hz)	CH	129.0	2.51 (m, H-7)	CH_2_	32.3
**1′**	-	C	133.1	-	C	133.5	-	C	133.4
**2′, 6′**	6.96 (d, *J* = 6.0 Hz)	CH_2_	130.5	7.03 (d, *J* = 8.5 Hz)	CH_2_	129.4, 129.0	6.99 (d, *J* = 8.5Hz)	CH_2_	130.4
**3′, 5′**	6.69 (d, *J* = 8.5 Hz)	CH_2_	116.3	6.69 (d, *J* = 8.5 Hz)	CH_2_	116.3, 116.3	6.69 (dd, *J* = 8.5Hz)	CH_2_	116.2
**4′**	-	C	156.7	-	C	156.8	-	C	156.5
**1″**	-	C	133.3	-	C	127.5	-	C	134.2
**2″, 6″**	6.98 (d, *J* = 6.0 Hz)	CH_2_	130.5	7.39 (d, *J* = 4.0 Hz)	CH_2_	130.5, 130.3	6.98 (d, *J* = 8.5Hz)	CH_2_	130.4
**3″, 5″**	6.70 (d, *J* = 8.5 Hz)	CH_2_	116.3	6.77 (d, *J* = 8.5 Hz)	CH_2_	116.9, 116.9	6.68 (dd, *J* = 9.0Hz)	CH_2_	116.3
**4″**	-	C	156.7	-	C	160.3	-	C	156.7

^1^H-NMR (500 MHz, CD_3_OD) δ, ^13^C-NMR (125 MHz, CD_3_OD) δ; Positions 1–7: heptane frame, Positions 1′- 6′ and 1″- 6″: 1,7-diphenyl frame.
